# Revisiting the Concept of Quietness in the Urban Environment—Towards Ecosystems’ Health and Human Well-Being

**DOI:** 10.3390/ijerph18063151

**Published:** 2021-03-18

**Authors:** Aggelos Tsaligopoulos, Stella Kyvelou, Nefta-Eleftheria Votsi, Aimilia Karapostoli, Chris Economou, Yiannis G. Matsinos

**Affiliations:** 1 Acoustic Ecology Laboratory, Department of Environment, University of the Aegean, 81100 Mytilene, Greece; economou@env.aegean.gr (C.E.); matsinos@aegean.gr (Y.G.M.); 2 Department of Economics and Regional Development, School of Sciences of Economics and Public Administration, Panteion University of Social and Political Sciences, 17671 Athens, Greece; kyvelou@panteion.gr; 3 Institute for Environmental Research & Sustainable Development, National Observatory of Athens, 15236 Athens, Greece; nvotsi@noa.gr; 4 School of Architectural Engineering, Democritus University of Thrace, 67100 Xanthi, Greece; akarapos@arch.duth.gr

**Keywords:** environmental noise, quietness, acoustic environment, soundscape, composite index, urban quiet

## Abstract

There is plenty of proof that environmental noise is a major pollutant in the urban environment. Several approaches were successfully applied for its calculation, visualization, prediction and mitigation. The goal of all strategy plans regards its reduction and the creation of quietness. This study aims to revisit the concept of quietness in the urban environment and attempts to portray a new understanding of the specific phenomena. “Quietness” as a term retains an ambiguity, and so far, it can be described as the lack of something, meaning the lack of noise that is portrayed by means of intensity. Several studies describe quietness as the combination of perceptual soundscape elements and contextual factors that can be quantified, combined, weighed and used as indicators of healthy soundscapes. In this research, the focus is on setting aside all indicators, either measuring the intensity or contextual ones and use solely quantifiable metrics regarding the acoustic environment, thus introducing a new composite index called the composite urban quietness index (CUQI). After testing the CUQI, in order to verify the results of previous research regarding the identification of quiet Areas in the city of Mytilene (Lesbos Island, Greece), the study concludes that CUQI is efficiently functioning even in this early stage of development.

## 1. Introduction

Simultaneous transmission of multiple sound sources is always happening in the urban environment [1]. This combined emission can cause increased intensity levels, which can be interpreted as noise. The negative impact of this pollutant on the physical and mental health of citizens [2], on the communicational efforts of the avifauna [3,4] and also on the quality of the overall environment [5,6,7] shaped the need to create and manage urban quiet areas [8].

The European 2002/49 Directive provides the definition of quiet areas in an agglomeration. An urban quiet area is meant to be “an area, delimited by the competent authority, for instance, which is not exposed to a value of L_den_ or of another appropriate noise indicator greater than a certain value set by the Member State, from any noise source”. L_den_ refers to the day-evening-night noise indicator used for overall annoyance assessment. The content of this definition offers an opportunity for further discussion regarding the concept of quietness. The debatable points of this definition come first of all, from the European Parliament’s suggestion for reducing intensity regardless of the sound source and from their recommendation on the delimitation of “quiet areas”. The concept of quietness contains an ambiguity that cannot be described using only a noise indicator that incorporates multiple artificial (anthropophony), biological (biophony) and also geophysical sound sources (geophony). Other physical, biological, psychoacoustical and contextual factors play an important role in sound perception [9]. Furthermore, the restriction of quietness in a single area is considered by the authors of this research as an unsustainable technique regarding the overall acoustic quality of a city.

Examining the concept of the cognitive landscape [10] within the soundscape context, we could conclude that acoustic/soundscape ecology attempts to contribute to environmental interpretation by introducing another dimension of information resulting from the soundscape [11]. The integration of several perspectives, such as sound-based information, individual-based perception, observer-based perception, and landscape characteristics, composes a network of interacting signals and signs [12]. The complex issue of quietness cannot be assessed through the use of a unique metric since it contains both physical and psychological attributes. Hence, there is an urgent need to investigate noise levels, the physical and mental health of citizens, the impacts on the fauna and the overall environmental quality under a combined and multiplex indicator of urban quietness. The development of a combined index composed of information elements could inaugurate a new era in soundscape research, but also in urban studies.

The term “quietness” is not included in the quiet area definition, probably due to its vagueness [13]. Quiet areas and quietness are kinship terms, but in a way, distinctive. Typically, quiet areas are the areas that are not affected by noise. They can simply be described as the areas that offer quietness; hence what is left to interpret is what quietness means. In this research article, we try to explore this issue and, furthermore, present the early development stage of a new composite index named composite urban quietness index (CUQI) that assesses aspects of quietness by not including the concept of intensity neither in a physiological nor in a perceptual way. Hence, in this study, we dare to deviate from all psychoacoustical terms similar to pleasantness, vibrancy, sharpness, and loudness [14,15,16] that are commonly used in order to describe a good soundscape, and we actually inaugurate a novel, integrated and strictly quantifiable index to assess the urban soundscape.

## 2. Scientific Background: Revisiting Quietness

### 2.1. Factors of Quietness

The acoustic environment and soundscape assessment initiatives could be distinguished into two general approaches: field noise surveys and laboratory tests, which in many cases are combined. In the first approach category (field noise surveys) we can distinguish: (i) interviews [17] (ii) social media data [18], (iii) soundwalks [19] and (iv) recordings [20]. In the second approach category (laboratory tests), we can identify: (v) analysis and listening tests [21], (vi) sound modeling [22], (vii) noise mapping [23,24,25], and (viii) soundscape simulations [26].

Quiet areas can be found in city parks, within building blocks, in courtyards, in gardens and in leisure areas. In rural areas, they often coincide with natural parks or protected areas, but they may also be part of an agricultural area or unused peri-urban land [27]. There are several selection criteria for an urban quiet area, including quantifiable metrics similar to a noise threshold and size and other not directly quantifiable but yet calculable attributes. These hard-to-measure criteria include concepts regarding the esthetic, cultural and visual dimension of an area, the effect of greenery on individuals and the urbanization rate [28]. Furthermore, psychoacoustical terms similar to vibrancy [14] are calculated in order to portray the way that an acoustic environment is being perceived. Qualities other than low noise levels, like, for instance, a safe and clean place or a pleasant view, are also well-received attributes of quiet areas. Following the above guidelines, in most cases, the resulted quiet areas refer to urban green spaces, excluding other types of places similar to public squares and walkways [29].

Furthermore, the creation of strategic noise maps is obligatory for every member state, but this is not the case for the identification and establishment of quiet areas. Therefore, only a few EU countries have established urban quiet areas. Additionally, there is no common approach regarding their identification, and each Member State follows a different path. These drawbacks have created inequalities at multiple scales [30].

Several auditory factors, including biological circadian/spatiotemporal phenomena similar to bird dawn chorus [31] and several non-auditory factors [32] similar to social [33], spatial [34], and structural factors [35], create distinctive acoustic environments. The above auditory factors, the soundmarks [36] and landmarks, contribute to the sonic identities [37] of areas and shape the way they are being perceived by individuals (aka the soundscape). The acoustic environment and, therefore, the soundscapes created can be measured, assessed, and compared using relevant indicators.

An acoustic indicator can be defined as a statistical metric that summarizes some aspects of acoustic energy distribution in a recording [38], while in some cases, several contextual and structural factors are incorporated. Similar to the classical ecological indicators, at least 28 different acoustic indicators have been recently proposed [39]. These indicators measure the range, smoothness, richness, or heterogeneity of an acoustic community in an acoustic environment [38,39].

The normalized difference soundscape index (NDSI) [40] evaluates the level of anthropogenic disturbance on the acoustic environment by calculating the ratio of biological sounds and human-generated sounds in an audio file. The aim of this index is the estimation of the anthropogenic disturbance in the acoustic environment, utilizing the fact that human-generated sounds range mainly between the frequency ranges 1–2 kHz, while biological sounds between 2 and 8 kHz. NDSI has a value range from −1 to +1, with +1 indicating that an audible signal contains only biological sounds. NDSI varies depending on the time and day of the recording and can be used to highlight sound differences over a time period. However, even a low value of the indicator may reveal the presence of biophony in the case of low-frequency signal transmission [41].

The acoustic complexity index (ACI) is based on the observation that biotic sounds, such as birdsongs, are characterized by a variability regarding intensity, while anthropogenic sounds, such as road noise, have constant intensity values. This index calculates the number of large peaks in terms of intensity in a spectrogram [42]. The majority of biotic sounds, unlike most anthropogenic sounds, have an inherent complexity. The long-term goal of ACI is to use it as a tool for extracting information from an acoustic environment in order to identify changes. Furthermore, it serves as a more efficient and faster monitoring tool regarding animal dynamics in an ecosystem [43].

At the same time, the acoustic diversity index (ADI), similar to the acoustic entropy index, uses the Shannon index to estimate acoustic diversity [44]. The same methods were used to calculate acoustic evenness index (AEI) [45], which is negatively correlated with ADI.

The physical, psychological, and contextual factors [9], the geometry of the surrounding buildings [46], the architectural contemporary and historical landscape characteristics [47], the ecological processes and other environmental factors formulate the acoustic environment and shape the soundscape. All the above information can be quantified, assigned with weights, and incorporated in a composite indicator in order to define aspects of the acoustic environment and attempt to describe the soundscape. Several indicators have been proposed. The quietness suitability index (QSI) (EEA Technical report No 4/2016) is based on the multidimensional character of the notion of quietness and is defined by quantitative data based on noise level measurements and qualitatively based on people’s perception regarding naturalness. Furthermore, the green soundscape index (GSI) [48] is based on the ratio of the perceived extent of natural sounds (PNS) and the perceived extent of traffic noise (PTN). A fuzzy approach to the creation of these equations/indicators is commonly used [49] with great results.

### 2.2. The Fuzziness of Quietness

The theory of fuzzy sets is a way of determining how well an object satisfies a vague description by assessing degrees of causality between concepts [50]. The verbal quantification of quietness and silence belong in this context. Silence is a circumstance where nothing is heard and could be correlated with the term “absence”. The absolute absence of sound is unavailable and can exist only as an abstract concept [51]. The possible polyphony of nature [52] and the cacophony of urban environments [53] may be absent under the circumstances, but even in an anechoic chamber, which is acoustically isolated, our own body generates sound [52]. Therefore, the phrase that something, or someone, or an area is quiet is vague. The intermediate stages of quietness, and the lack of an absolute state of silence, support this uncertainty.

In order for the above logic to be understood, an example is given. A hypothetical acoustic environment can be characterized by environmental noise levels of a specific indicator of 56 dB (A), while another of 55 dB (A). According to the World Health Organization (WHO), but also according to classical logic, the second sound environment (55 dB) is quieter. Fuzzy logic, however, states that the above proposition is true, but with some, not absolute, degree of truth. Fuzzy cognitive mapping (FCM) can be described as a qualitative modeling procedure of system operation [50,54,55,56] that can aid the process of identifying the factors that cause the state of quietness.

### 2.3. Combining the Practical and the Pleasurable Benefits of Quietness

There is a growing need to create good soundscapes deriving from acoustic environments that promote physical and psychological health [57]. Pleasantness is one of the main factors [22] in describing a good soundscape, and for this matter, urban park visitors have a preference for natural sound [52,58,59]. It is understood that the soundscape is affected by landscape factors [60] hence; soundscape planning and design could possibly depend on landscape interventions. The authors of this research paper stress the need for soundscape planners to seek ecological co-benefits in their planning and combine pleasantness with practical and ecologically viable long-term benefits regarding climate change adaptation [61] and adopting green infrastructure and nature-based solutions (NbS) in the urban environment [62].

Climate issues retain a level of uncertainty. Statistical uncertainty (also referred to as “noise” in a metaphorical sense) raises concerns about the alterability of nature regarding numerous unpredicted changes in species distribution and abundance [63]. Uncertainty refers to the improbability surrounding a variable when its state at a specific point is unknown, but the probability distribution that characterizes that variable is known [64]. For example, the probability of an unexpected noise event occurring in an otherwise quiet acoustic environment is a form of statistical uncertainty.

Uncertainty in climate issues [65] is supported by two different schools of thought, the experiential and analytic processing systems [66]. The analytical process includes a mechanism that relates current situations with the combination of past experiences, making it easier to use statistical concepts. Nevertheless, the analytical process has been shown to be an inhibitory factor regarding immediate decision-making [67]. Experiential processing relates present situations with experiences. The human mind does not directly react to threats that seem to occur in the distant future. As a result, long-term distresses such as climate change do not affect as much as more immediate problems. The experiential context of noise is the final stage of a general multifold problem directly affecting human wellbeing and ecosystem health. The use of this experience in terms of tackling a combination of problems could provide an immediate solution.

The soundscape refers to the personal experience of an acoustic environment and, therefore, a phenomenological issue. Phenomenology is a school of thought that focuses on experience [68]. Consequently, the perception of sounds is a personal experience that is difficult to convey and describe. Hence, far, the averaging of perceptual responses has provided comparable data to be incorporated in indicators, decision-making and soundscape planning [69]. However, the study of an acoustic environment is a phenological issue. Phenology is described as “the study of the timing of recurrent biological events, the causes of their timing with regard to biotic and abiotic forces, and the interrelation among phases of the same or different species” [70].

The combination of different opinions using psychoacoustical metrics can provide valuable data regarding the assessment of soundscapes using a phenomenological approach and an experiential processing system. The assessment of acoustic as a phenological matter could be achieved using an analytical processing system that utilizes metrics related to the physical aspects of sound produced and propagated in a landscape. Therefore, decision-making regarding soundscapes and acoustic environments should include both experiential and analytic processing systems in order to combine practical and desirable outcomes. Due to the fact that natural sounds promote pleasantness and create good soundscapes [59], an opportunity for sustainable acoustic environment planning and design is at hand. Therefore, problems of the present and of the future could be tackled by promoting nature-based solutions with the co-benefit of pleasantness and climate change adaptation through the medium of quietness.

### 2.4. The Equity in Quietness

Quietness could be considered as an ecosystem service attributed to urban green areas [71] being both a cause for and an outcome of a healthy ecosystem. The co-benefits of quietness that expand from human wellbeing to environmental health and climate change adaptation are undeniable [61]. It is well understood that if urban sounds are perceived as a negative factor, the need for quietness is higher, but is perceived as a positive factor, in terms of liveliness and vibrancy, the specific necessity is reduced [72]. Most likely, the negative perception of urban sounds is subjective to several socioeconomic factors similar to the higher unemployment rate [73].

The interdisciplinary field of acoustic and soundscape ecology, along with the theoretical broadening of the term soundscape, paved the way for new approaches regarding noise management [74]. Soundscapes are a vital part of the sonic identity of a city [75], and quietness as an attribute and part of this identity [31,68] is an issue that needs to be clarified.

Environmental noise is a serious multifactorial problem for both human wellbeing and biodiversity [13]. Noise as a part of the sonic identity of a city, or at least in some neighborhoods of a city, has both subjective and objective elements [76]. Schafer stated that noise could be viewed as an unwanted sound highlighting in this way its perceptual characteristics. Furthermore, noise can be considered as an unmusical sound (non-periodic vibration) and any loud sound that could possibly mask or intercept signals [53,75]. The latter “tangible” attributes of noise have led to the creation of a quantifiable metric as a way to communicate the sound intensity, and therefore, to be used as a noise abatement index. The decibel scale served well for this matter, but dBA is not well correlated with human perception [9].

These concepts presuppose the absence of a quantity, the quantity of noise. In addition, the concept of noise is a subject of ambiguity regarding its definition and perception. In some cases, noise coincides with its psychoacoustic usage as an unwanted sound. In this case, noise is not an absolute state but is a subject of the listener’s perception and hearing capabilities. Additionally, noise concerns all cases of persistent emission of acoustic energy, regardless of the source and the meaning of the sound produced. Therefore, the simultaneous, bipolar contribution of a source to the signal-to-noise ratio is possible.

A different approach to the problem of noise is that it can be considered as a class issue in an economic sense. Noise may be considered an insignificant problem in relation to others, which concerns “insignificant” people regarding class and status [77]. In this sense, if noise is a problem concerning the lower class citizens, then quietness is a benefit of the “elite”. It is well understood that quietness should be considered a public good, and it should be treated accordingly, keeping in mind sustainability and equality [13].

Analyses of environmental inequalities show that across the whole urban territory, income is a robust interpreter for noise exposure [78]. Several studies have also observed positive associations between road-traffic noise and deprivation [79], identifying a clear deficiency of environmental equity on the Island of Montreal. Their research indicated that disadvantaged groups bear a double burden of higher exposure to noise and low-income status. Their findings showed that noise exposure was strongly correlated with socioeconomic indicators, such as median household income, percentage of people who live in non-affordable housing—spending over 30% of their income on housing, percentage of people below the low-income line and with a social deprivation index combining several socioeconomic variables. Similar outcomes are identified by [80] associating noise levels and household income, median household value, the proportion of non-white residents, and the percentage of the young population.

Another research outcome is the positive association between depressed mood and high road traffic noise exposure in residential settings, which is happening for all urban citizens independently from ethnic minority or socioeconomic status [81]. Moreover, depreciation of land and property values is also resulting from environmental noise. Several studies have been conducted to assess the loss of rental income due to noise. In Switzerland, a finding is that almost 60% of the calculated costs of noise (more than 1 billion Swiss francs per year) correspond to losses of property value, caused primarily by road noise, which is the highest volume noise in the country [81]. Buildings exposed to excessive noise levels have lower rent or sale prices compared with those located in quiet areas.

Quietness seems to be the panacea regarding the desired relief from noise pollution, but still, as a term, needs to be clarified in order to be properly used in planning. The association of quietness with silence is common. Recent COVID-19 related restrictions in everyday life have portrayed a different view on this matter [82]. The profound silence in otherwise noisy or acoustically vibrant cities could possibly be associated with a sense of sensory deprivation, anxiety due to confinement and health implications [83], but has proven to be beneficial for songbirds that reclaimed favored frequencies [76,84], but also, in some cases, for other wildlife species [77]. Therefore, the benefits of quietness should be differentiated from the implications caused due to silence.

## 3. Materials and Methods

### 3.1. The Composite Urban Quietness Index

Urban quiet areas can be described as the areas that, even though noise is not absent, it is not dominant [78,85]. In order to explain and quantify the aforementioned physical but yet fuzzy condition, a multi-fold approach with the use of existing indicators is required. However, several inconsistencies among indicators produce results that are hard to decipher. Consequently, the identification of the appropriate indicators and their combination in a new composite indicator [86] is presented below.

In the current research, data used were collected from previous research regarding the identification of Mytilene’s quiet areas, which is a city located on the island of Lesbos (North Aegean, Greece) [87]. Noise measurements and sound recordings were also collected and analyzed in order to extract intensity and spectral indices.

Prior to the noise measurements, the sound level meter PRO-DX of Castle Group was calibrated using the standard 94 dB calibrator, as is required for all class 1 measurement instruments according to the EN61326-1:1997 + A1:1998 specifications. A sampling protocol was created, and data regarding the Leq noise level were collected. The protocol involved 10 min noise measurements and sound recordings in all case study areas chosen. Strategic positioning during the measurements, considering the size and the topography of the area, resulted in a realistic outcome. The spots were chosen in order to capture the whole extend of the area, using open spaces far from high walls or sharp urban structures. The idea was to keep the measurements unaffected by factors similar to sound reflection caused by the structural characteristics of the area. Finally, each 10 min sampling was carried out at the height of 1, 5 m above the ground. Furthermore, the Tascam DR-2d portable digital high-resolution recorder was used in order to obtain soundscape recordings. The sound files collected were processed in order to determine the ACI [43] and the NDSI [41] using the R statistics v. 3.1.3 software (R: The R Project for Statistical Computing) and the associated packages Seewave, TuneR, Ineq and Soundecology [88,89]. In order to extract the most important variables (indicators) from the large data pool created and use them as sub-indicators for the composite urban quietness index, we used the principal component analysis method.

### 3.2. Principal Component Analysis of Acoustic Environment Factors

**Figure 1:**
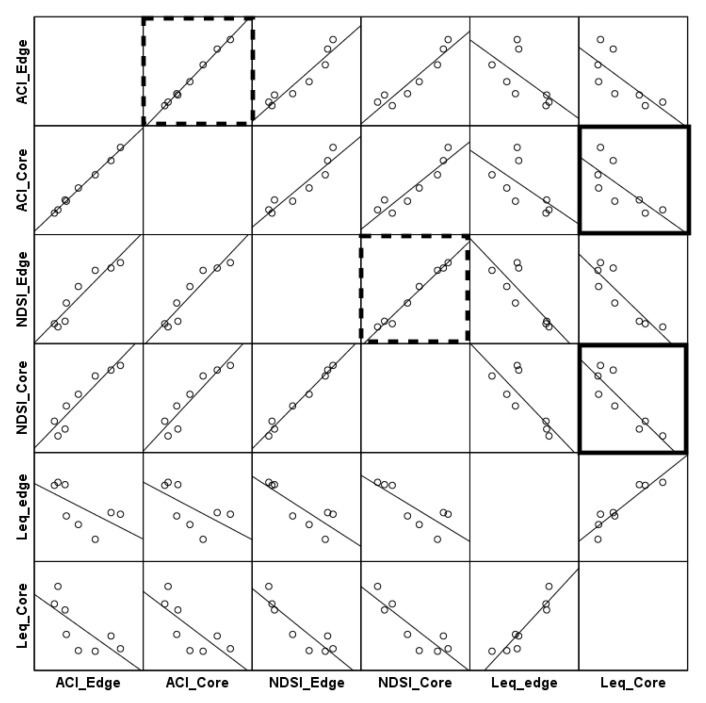
The correlation matrix highlighting several negative correlations and several positive correlations represented in dashed border lines.

According to several studies, a low-intensity level is an important but yet insufficient factor that characterizes a quiet acoustic environment [9,13,87]. The statistical analysis on the data collected and, more specifically, the Spearman’s rho correlation conducted among the acoustic indicators ACI, NDSI and the L_eq_ noise indicator highlighted the negative effect of noise on complexity. Several strong negative correlations among the ACI and Leq indicators resulted through the analysis supporting this hypothesis (rs = 0.762, *p* = 0.028). Simultaneously, strong positive correlations among the acoustic indicator’s levels on the area’s edges and core resulted through the analysis (rs = 0.976, *p* = 0.000). A more detailed representation of the correlated and non-correlated variables extracted from the sound recording and noise level measurements is presented in Figure 1.

**Figure 2:**
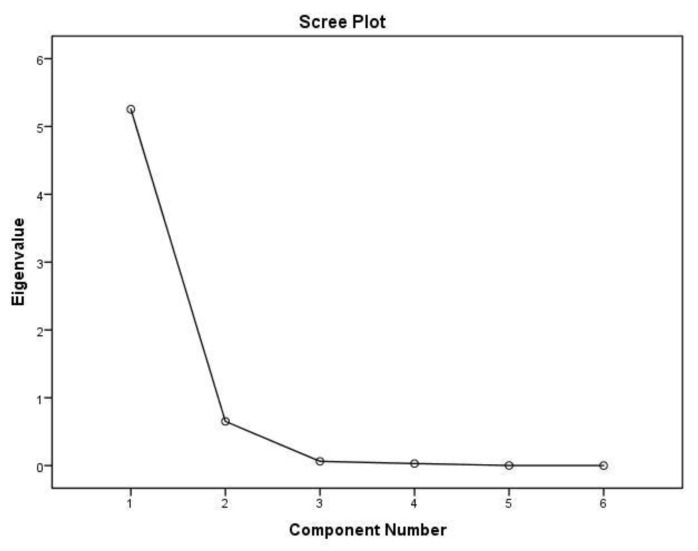
Scree plot indicating the number of factors to be extracted.

**Figure 3:**
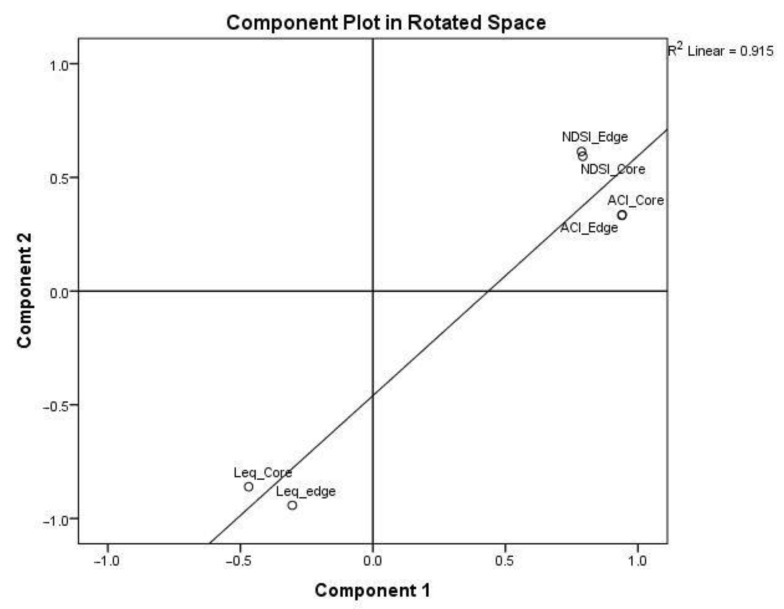
Component plot in rotated space.

In order to identify the factors that contribute to the state of quietness, a principal component analysis was conducted (KMO = 0.655, Bartlett’s test of sphericity *p* = 0.000) using the ACI levels, the NDSI levels and the L_eq_ levels on each area’s edges and core. Using the resulted scree plot (Figure 2), two principal components were extracted by the PCA. These principal components explain a total of 98% of the variance with high positive loadings. Furthermore, the important factors in each component were identified using the orthogonal varimax rotation with Kaiser normalization (Figure 3).

Component 1 explains the 87% of the variance with high (>0.40) loading for the ACI levels on the area edges and core and could represent complexity. Component 2 explains the 10% of the variance with positive loading on the NDSI levels on the areas edges and core and could represent intensity.

**Figure 4:**
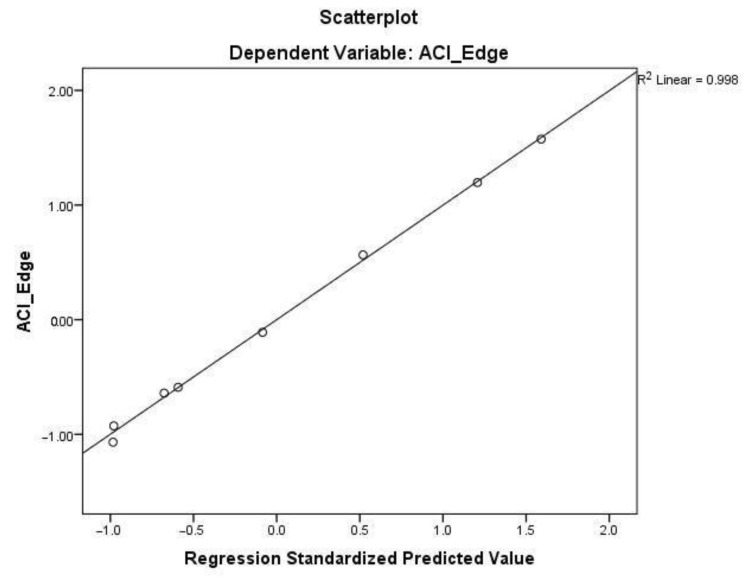
Regression standardized predicted value of acoustic complexity index levels of the area’s edges.

In summary, the acoustic complexity indicator is an important factor that determines the principal components, while the Leq levels present strong negative correlations. Simple linear regression was carried out to investigate the relationship between acoustic complexity index levels of the area edges, with component 1 represented as complexity. As it can be seen in Figure 4, the scatterplot showed that there was a strong positive linear relationship between the two, which was confirmed with a Pearson’s correlation coefficient of 0.999. Simple linear regression showed a significant relationship between ACI and the new complexity variable (*p* < 0.001). The slope coefficient for complexity was 0.941. The R^2^ value was 0.998, so 99.8% of the variation in ACI levels can be explained by the model containing only complexity. The scatterplot of standardized predicted values versus standardized residuals showed that the data met the assumptions of homogeneity of variance and linearity, and the residuals were approximately normally distributed.

### 3.3. CUQI Rationale and the Definition of Quietness

It is undeniable that quiet and green areas in an urban environment are beneficial, but traffic noise impedes the stress recovery offered [57,90]. This leads to noise assessment using traditional metrics similar to noise indicators, with doubtful results [9]. Recent findings suggest that the spectral dimension of sound is a more efficient way regarding the prediction of soundscapes [59]. Complexity is an ecological term used to describe the state of an ecosystem and is linked to other ecological concepts similar to resilience, integrity and diversity [91]. Due to the symbiotic relationship of quiet areas and biodiversity, ACI was chosen as the main quiet area descriptor. As mentioned above, ACI is based on the observation that biotic sounds are characterized by numerous intensity peaks in contrast to anthropogenic sounds that are constant [42]. Subsequently, high values of ACI indicate a high amount of biophonic sounds, hence, increased levels of biodiversity. However, the unpredicted behavior of sounds in an urban environment, regarding their production and propagation, could result in several misinterpretations of anthropophony and biophony. Therefore, ACI should be accompanied by an additional sub-indicator that can outline which category of sound (anthropophony or biophony) prevails. The NDSI could pose as an ideal descriptor of an acoustic environment, highlighting the circumstances that anthropogenic sound sources prevail.

When it comes to the creation of a healthy urban acoustic environment or a quiet area, several recipients must be taken under consideration, including human wellbeing and environmental sustainability promoting the symbiotic relationship of quiet areas and biodiversity. A strictly anthropocentric planning approach calls for a perceptual assessment with information collected by persons [69], using descriptors similar to vibrancy [25]. Therefore, the use of preferences for soundscape design [92] can eventually lead to acoustic environment planning that has a direct effect on the way that is being perceived, aka the soundscape [93]. The planning of quiet and green areas includes other concepts similar to ecological connectivity. The fact that increased intensity levels pose a non-physical barrier [83] that impedes ecological connectivity should be taken under consideration.

Taking into account the above information, a definition of urban quietness is provided below:

Urban quietness regards a balanced public acoustic environment where complex sounds prevail regardless of intensity and source, created or preserved in the context of environmental sustainability and environmental equity.

### 3.4. CUQI Data Collection Protocol

**Scheme 1:**
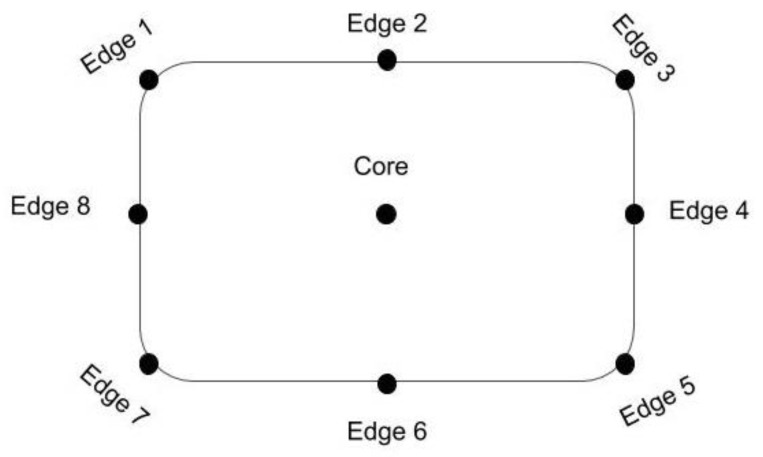
A hypothetical orthogonally shaped area indicating the 8 sampling points at the edges and the one at its core.

The provision of a specific sampling protocol is of vital importance. The proposed sampling protocol attempts to highlight the fluctuations of acoustic complexity among the edges of an area to its core using short-term sound recordings. So far, a specific type of area is considered in the sampling protocol shaped roughly as the hypothetical area presented in Scheme 1. The acoustic complexity values at eight surrounding sampling points located on the area’s edge and the value of one point located on the area’s core can be calculated (Scheme 1) using sound recordings.

The concept supporting CUQI includes the subject of acoustic balance on the spatial extent of the potential quiet area. The concept of balance refers to the analogy of a quantifiable metric, which in this case is ACI. The averaged value of the acoustic complexity data from the area’s edges and the value derived from the area’s core (Scheme 1) provides the ratio of the complexity balance (CB) index.

The CB ratio (1) is calculated as:

(1) $$\mathrm{CB}=\;\frac{\bar{e}}{c}$$

where:

ē = the averaged complexity values obtained from the area’s edges;

c = the complexity value obtained from the core of the area;

A ratio of CB close to 1 indicates an equally distributed and well-balanced acoustic environment in relation to its acoustic complexity. If the ratio of CB is higher than unity, then the area’s core is poor in complexity, indicating lacking factors that increase the acoustic vibrancy. If the CB ratio is less than unity, then the area’s edges are probably affected by masking noise. In a similar fashion to the signal-to-noise ratio, the CB ratio alone could be used as a decision-making tool regarding acoustic environment management.

In order to highlight the degree of complexity, the range of the acoustic complexity values (*RG_ACI_*) for all sampling points is calculated. The *RG_ACI_* (2) is calculated as:

(2) $$RG_{ACI}=\;ACI_{max}-ACI_{min}$$

where:

*ACI_max_* = the maximum acoustic complexity value derived from all nine sampling points, including the core;

*ACI_min_* = the minimum acoustic complexity value derived from all nine sampling points, including the core.

The determination of whether anthropogenic sounds prevail in the acoustic environment is designated through the extraction of *NDSI*. The occurrence of anthropogenic disturbance (*AD*) in the acoustic environment is incorporated by using a fraction containing the averaged *NDSI* values to the absolute value of the same result. The goal of the *AD* ratio is to keep the possible negative sign of *NDSI* in case of human noise dominance without affecting the outcome. The *AD* ratio (3) is calculated as:

(3) $$AD=\frac{NDSI}{\left|NDSI\right|}$$

where:

*NDSI* = averaged value of the normalized difference soundscape index derived from all nine sampling points, including the core;

|*NDSI*| = the absolute value of the averaged *NDSI* outcome.

The above Equations (1)–(3) are incorporated in one composite index called the composite urban quietness index (4):

$$CUQI=\;\frac{NDSI}{\left|NDSI\right|}\times \;\left(\left(ACI_{max}-\;ACI_{min}\right)\times \frac{\bar{ACI_{edge}}}{ACI_{core}}\right)$$

expressed in short as:

(4) $$CUQI=AD\times \;\left(RG_{ACI}\times CB\right)$$

where:

*AD* = anthropogenic disturbance;

*RG_ACI_* = range of the acoustic complexity values;

*CB* = ratio of the acoustic complexity values.

A positive high *CUQI* score indicates an equally balanced acoustic environment of high complexity. A negative signed low leveled UQI score indicates an ununiformed acoustic environment of low complexity where anthropogenic and probably masking sounds prevail.

### 3.5. Testing the CUQI in Mytilene (Lesbos Island–Greece)

**Figure 5:**
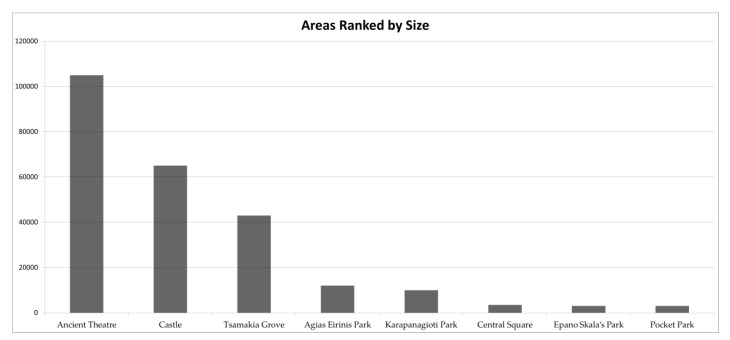
Potential quiet areas ranked by size measured in square meters.

Recent research related to urban quiet areas has produced a flexible protocol regarding the identification of quiet areas in an urban context. By utilizing local knowledge and citizen science techniques, a number of acoustic environments worthy of investigation derived. The 8 case study areas are presented below and are ranked by size in Figure 5.

#### 3.5.1. Case Study Areas

##### Tsamakia Grove

The Tsamakia Grove is an urban green space located in the suburbs of the city of Mytilene, which is the capital of Lesbos Island located in the North Aegean Region. The specific grove is about 30.000 m^2^ and mainly consists of pine trees (*Pinus* sp.) with numerous bird species, including large amounts of hooded crows (*Corvus cornix*), common blackbirds (*Turdus merula*), great tits (*Parus major*) and other similar species. It is a place used regularly by local people and tourists for leisure and exercise, while it is a part of the local road network, but with limited access. Finally, Tsamakia is considered to be a candidate urban quiet area, as highlighted in previous research regarding the identification of quiet areas in agglomerations [87,94].

##### Pocket Park

A park located in the city center was incorporated in this study. Its rather small size constitutes this place like a pocket park. A scarce amount of plant and bird species are present. It is a place used regularly by local people.

##### Epano Skala Park

The Epano Skala Park is a small urban park used as a playground and a dog park. On several occasions, small in scale festivals take place, especially during the summer season.

##### Agias Eirinis Park

The Agias Eirinis Park is a small urban green area located in the center of Mytilene. This park is approximately 12,000 m^2^ and mainly consists of false acacias (*Robinia pseudoacacia*) and other deciduous trees and shrubs. In the specific urban green space, numerous songbird species can be found with great seasonal variations. Hooded crows (*Corvus cornix*), common blackbirds (*Turdus merula*), great tits (*Parus major*), common chiffchaffs (*Phylloscopus collybita*), European robins (*Erithacus rubecula*) and other similar species are nesting there. Furthermore, it is a highly visited public green space due to the fact that it contains a cafeteria, a playground and a church. It is surrounded by streets of high volume traffic; however, depending on the season, the foliage of the false acacias (*Robinia pseudoacacia*) acts as a noise barrier by slightly reducing the noise impact.

##### Karapanagioti Park

Karapanagioti Park is another small urban green area located in the city’s center close to Agias Eirinis Park. It is approximately 9500 m^2^, and it consists of mulberries (*Morus alba*), false acacias (*Robinia pseudoacacia*) and other tree species and large shrubs similar to bay laurels (*Laurus nobilis*). Several bird species can be found in this green area, among which Eurasian jays (*Garrulus glandarius*), hooded crows (*Corvus cornix*), common blackbirds (*Turdus merula*), great tits (*Parus major*), Eurasian blue tits (*Cyanistes caeruleus*), common chiffchaffs (*Phylloscopus collybita*), European robins (*Erithacus rubecula*) and white wagtails (*Motacilla alba*). This green space is also surrounded by streets of high volume traffic, and furthermore, it is located next to Mytilene’s soccer stadium. Consequently, Karapanagioti Park is affected by both environmental noise from the surrounding road network and artificial light pollution emanating from the soccer stadium’s headlights, reaching as high as 200,000 lumens per fixture.

##### Central Square

The Central Square is a public space of approximately 1000 m^2^ located in the center of Mytilene. It is a highly visited place by Mytilene’s inhabitants and tourists. Several activities take place there, similar to protests for political reasons and concerts, making Sapfous Square among the most important and popular public spaces. Apart from some cosmetic shrubs, there is no vegetation there, and furthermore, apart from domestic pigeons (*Columba livia domestica*), no bird species can be found or heard.

##### Ancient Theater

The ancient theater is both an archeological site and a green area. It is approximately 125,000 m^2^, and it is located at the highest point of the city’s outskirts. It mainly consists of pine trees (*Pinus* sp.) with several bird species, including hooded crows (*Corvus cornix*), common blackbirds (*Turdus merula*).

##### Castle

The Byzantine castle is a highly visited archeological site and a green area located in Mytilene’s outskirts on the east side. It is approximately 265,000 m^2^ and mainly consists of pine trees (*Pinus* sp.) with numerous bird species, including large amounts of hooded crows (*Corvus cornix*), common blackbirds (*Turdus merula*), great tits (*Parus major*) and other similar species. The Byzantine castle consists of several subareas, including small beaches, a smaller lighthouse, groves and monuments. It is also a highly visited place for various reasons, among which relaxation, recreation and outdoor sports.

**Scheme 2:**
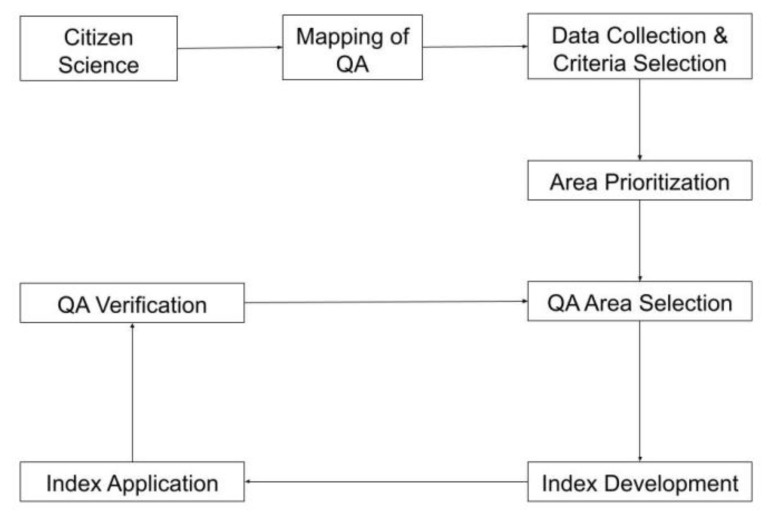
The previous workflow chart.

Summing up, several criteria were used in order to identify the quiet areas of Mytilene. Acoustical criteria that refer to noise thresholds, urban functionality criteria, the type of land cover, the degree of vegetation, the health restorative elements, the size of the area, the visual qualities and the way it is being perceived are all criteria of selection. With the aid of a multiple-criteria decision analysis tool, the potential quiet areas of the city under consideration were highlighted and ranked. This workflow chart is presented in Scheme 2.

It was concluded that noise limitation using the decibel scale is not the most important feature of quiet areas or a major criterion regarding their identification. It is well established that the interdisciplinary collaboration of ecologists and architects/urban designers operating in an acoustic and soundscape ecology framework could produce schemes that involve action plans regarding noise mitigation and, more specifically, regarding the structural and functional connection of urban quiet areas. In previous research regarding the ecological connectivity of urban green, quiet areas [95], the non-physical barrier imposed by noise intercepting the otherwise equally distributed levels of acoustic complexity was underlined.

## 4. Results

### Verification of Quiet Areas Using CUQI

**Figure 6:**
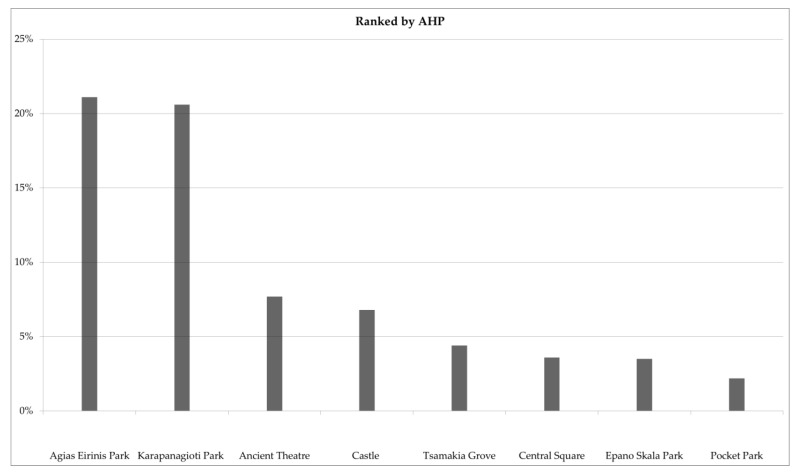
The potential quiet areas in order based on the results of the Analytical Hierarchy Process conducted (Matsinos et al., 2017).

**Figure 7:**
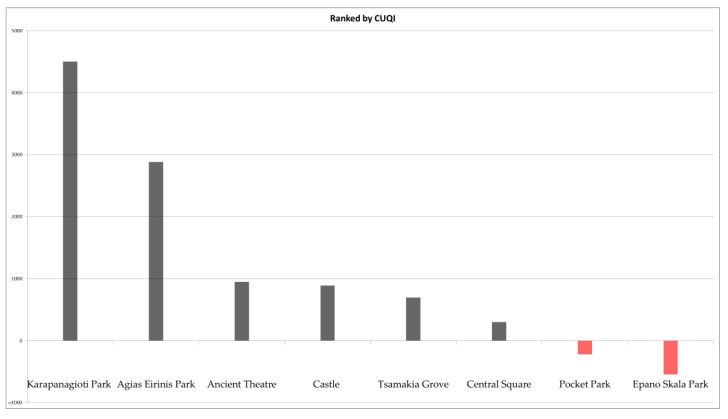
Ranked quiet areas using the composite urban quietness index.

The outcome of previous work regarding quiet area prioritization resulted in a hierarchy of the possible choices. In Figure 6 the ranked options are presented. The Agias Eirinis Park and the Karapanagioti Park were the two top choices. The CUQI extractions regarding the same areas gave similar results with small alterations regarding the order of the first two areas and of the last two (Figure 7).

The eight similarly shaped case study areas were assessed using the previously published protocol and now analyzed using CUQI. Therefore, a hierarchical descent is visible (Figure 6 and Figure 7), beginning from the areas that are most likely to be “quiet areas”, leading to the ones that are not. Future changes that regard the structural composition of the areas studied may alter these results. Furthermore, due to the fact that acoustic environments and, therefore, soundscapes are dynamic and subject to constant change, diurnal and seasonal variations will probably alter the results.

## 5. Discussion

Results provided by the calculations of CUQI seem to give very similar results with the previous protocol used for the identification of urban quiet areas. CUQI seems to comply with research requirements balancing between the multifactorial perspectives of environmental complexity as an easy-to-implement decision-making tool.

Noise indicators similar to L_eq_, along with the dB(A) scale, have faced criticism [9] regarding their effectiveness. The statistical analysis conducted highlighted the principal factors that define quietness, and the Leq indicator did not meet the standards. Even though metrics that regard intensity is very important factors for soundscape and acoustic environment assessment, they are not sufficient to describe the concept of quietness.

According to the principal component analysis conducted, ACI seems to be a good metric for quietness description. Nevertheless, it is expected that the diurnal patterns occurring in an acoustic environment will alter the sub-indicators results and, therefore, the CUQI index outcome. Future research involves the analysis of areas with different shapes in order for the CB sub-indicator to be spatially expanded.

## 6. Conclusions

The new proposed definition of quietness provided by the current research is expected to shed more light on this vague issue and broaden the term of quietness, essentially differentiating it from silence. People’s preferences in natural sounds should be viewed as an opportunity for sustainable development. The specific demand of urban park users has created a new market regarding the creation and promotion of good soundscapes. This research introduced a new, ecological approach in quietness able to provide urban planners, architects and sound designers a new tool in soundscape planning, with numerous co-benefits regarding sustainability. The existence of a common conceptual framework regarding quiet areas, and the collaboration among ecologists, urban planners/designers and architects, working under the aegis of acoustic ecology, may build the foundation of a new era regarding a truly equitable and sustainable practice.
